# *In Vivo *Induction of Apoptosis by Fucoxanthin, a Marine Carotenoid, Associated with Down-Regulating STAT3/EGFR Signaling in Sarcoma 180 (S180) Xenografts-Bearing Mice

**DOI:** 10.3390/md10092055

**Published:** 2012-09-20

**Authors:** Jun Wang, Shihui Chen, Shiqiang Xu, Xing Yu, Dongqing Ma, Xiamin Hu, Xiaolu Cao

**Affiliations:** Department of Pharmacology, College of Medicine, Wuhan University of Science and Technology, Wuhan 430065, China; Email: wangj79@hotmail.com (J.W.); chenshihui@2008.sina.com (S.C.); coolviewer@163.com (S.X.); yuxinglovely@163.com (X.Y.); dongjing20082009@163.com (D.M.); caoxiaolu1126@163.com (X.C.)

**Keywords:** fucoxanthin, antitumor, epidermal growth factor receptor, signal transducers and activators of transcription 3, marine natural products

## Abstract

Previous *in vitro* researches have showed that fucoxanthin, a natural carotenoid isolated from sargassum, can inhibit proliferation or induce apoptosis in human neuroblastoma, hepatoma, leukemia, colon carcinoma, prostate cancer or urinary bladder cancer cells. But the precise mechanism by which fucoxanthin exerts anticarcinogenic effects is not yet fully understood. In this study, we performed an *in vivo* study to investigate the anti-tumor effect and mechanisms of fucoxanthin on xenografted sarcoma 180 (S180) in mice. Results revealed that fucoxanthin significantly inhibited the growth of sarcoma at the dose of 50 or 100 mg/kg. TUNEL analysis showed that the number of positive cells in the fucoxanthin-treated group was higher than that in the control group. Western blotting analysis also revealed the suppressed expression of bcl-2 and enhanced expression of cleaved caspase-3 by fucoxanthin. In addition, immunohistochemistry analysis and Western blotting analysis showed that fucoxanthin significantly decreased the expressions of survivin and vascular endothelial growth factor (VEGF). Most importantly, fucoxanthin inhibited the expressions of the epidermal growth factor receptor (EGFR) and STAT3 and phosphorylated STAT3 proteins. These results indicated that *in vivo* induction of apoptosis by fucoxanthin is associated with down-regulating STAT3/EGFR signaling in S180 xenografts-bearing mice.

## 1. Introduction

Fucoxanthin is an oxygenated marine carotenoid available in different types of edible seaweed such as *Laminaria japonica*, *Undaria pinnatifida *and *Hijikia fusiformis* [1,2]. This compound has pharmaceutical properties, such as anti-obesity [3], anti-mutagenicity [4] and anti-inflammation [5]. However, the anti-cancer effect of fucoxanthin has been mostly focused [6], and fucoxanthin has become a prospective substance to be developed as a pharmaceutical antitumor agent [7]. Previous *in vitro* researches have showed that fucoxanthin can inhibit proliferation or induce apoptosis in human neuroblastoma [8], hepatoma [9,10,11], leukemia [12,13,14], colon carcinoma [15,16], prostate cancer [10,17] or urinary bladder cancer [18] cells. The antitumor effect of fucoxanthin has been demonstrated to be mediated through the up-regulation of p21WAF1/Cip1 [15] and ROS-mediated bcl-xl pathway [14], the down-regulation of cyclin D [11] and associated with GADD45, p38 MAPK or SAPK/JNK [10,17]. In our previous study, we have also found that fucoxanthin downregulated the expressions of cyclinB1 and survivin, induced cell cycle arrest in G2/M phase and apoptosis in human gastric adenocarcinoma MGC-803 cells [19]. In addition, the reduction of cyclinB1 by fucoxanthin was associated with JAK/STAT signal pathway [19]. Thus, fucoxanthin is believed to have multiple mechanisms to prevent the growth of tumor cells [20]. However, the precise mechanism by which fucoxanthin exerts these anticarcinogenic effects is not yet fully understood.

The signal transducers and activators of transcription (STATs) and epidermal growth factor receptor (EGFR) are commonly expressed and activated in many malignancies. STATs activate selected genes involved in oncogenesis, and EGFR is an upstream activator of several signaling pathways involved in tumor progression [21]. STATs comprise a family of seven structurally and functionally related proteins. Aberrant activation of STAT3 is commonly observed in tumors and is strongly associated with tumor development and progression [22]. STAT proteins participate in tumorigenesis through up-regulation of genes encoding apoptosis inhibitors (myeloid cell leukemia sequence 1 (MCL1), BCL2-like 1 (BCL2L1)) and cell-cycle regulators (cyclin D1/D2, MYC) [23]. STAT3 is also involved in tumor progression through inducing angiogenic factors, such as vascular endothelial growth factor (VEGF) [24]. EGFR is one of four homologous transmembrane proteins that mediate the actions of a family of growth factors including EGF, transforming growth factor-α, and the neuregulins [25]. Elevated expression of EGFR and/or its ligands is common in many types of epithelial cancer, and such change has been shown to be an important component for maintaining the proliferative capacity of the tumor cells [26]. Moreover, recent studies have revealed a critical role of STAT3 in maintaining EGFR-mediated cancer cell proliferation [27,28]. And EGFR likely activates STAT in a manner distinctive from other mechanisms of STAT activation [21]. In view of the important role of STAT3/EGFR signaling in tumor development and progression, the methods to inhibit EGFR in conjunction with oncogenic STATs may represent a novel and attractive therapeutic strategy for cancers characterized by upregulation of EGFR signaling [21]. In the previous study, we have demonstrated that the expressions of survivin and STAT3 genes were regulated by fucoxanthin [19]. However, the effect of fucoxanthin on EGFR/STAT3 signal pathway is still unclear.

In addition, almost all previous studies on the anti-cancer effect of fucoxanthin were based on *in vitro* experiments, and there have been very few systematic studies of *in-vivo* activity. Actually, the amount of EGFR expressed on mouse S180 is abundant [29]. In an effort to evaluate the overall anti-tumor effect of fucoxanthin and its molecular mechanism, we have performed an *in vivo* study in S180 tumor-bearing mice. These *in vivo* results are consistent with our *in vitro* results showing a potent induced effect of fucoxanthin on the apoptosis of tumor, which is associated with EGFR/STAT3 signal pathway.

## 2. Results and Discussion

### 2.1. The Effect of Fucoxanthin on the Tumor Growth of S180 Sarcoma in Mice

As shown in Table 1, fucoxanthin (at the dose of 50 and 100 mg/kg) caused a significant decline of sarcoma weight in a dose-dependent manner compared with the model control group (*p* < 0.05 or *p* < 0.01). The tumor inhibitory rate of fucoxanthin was 41.0% and 72.2% at the dose of 50 and 100 mg/kg, respectively. 

Our *in vivo* study provides information that fucoxanthin reduced the S180 sarcoma weight in a dose-dependent manner indicating its exact role in anticancer therapy, which appears consistent with previous *in vitro* researches [8,9,10,11,12,13,14,15,16,17,18,19]. Fucoxanthin at the high-dose showed better suppressive effect on the tumor growth of S180 sarcoma than that of cyclophosphamide (CTX). Moreover, during the experimental period, we found that some mice in CTX-treated group were in bad mental state, manifested as sluggishness and drowsiness, with thinning of hair (data not shown), which was not found in the fucoxanthin-treated groups.

**Table 1:** Effect of fucoxanthin on the tumor growth of sarcoma 180 (S180) in mice (mean ± SD, *n* = 15). Fucoxanthin was given by gavage for 3 weeks at the following doses: 25, 50, 100 mg/kg and the mice in positive control group were administrated with CTX (20 mg/kg) once per day.

Group	Dose (mg/kg)	Tumor weight (g)	Tumor inhibitory rate (%)
Model control	—	1.07 ± 0.66	—
Fucoxanthin	25	0.81 ± 0.61	24.4
Fucoxanthin	50	0.63 ± 0.25 *	41.0
Fucoxanthin	100	0.30 ± 0.19 **	72.2
CTX	20	0.38 ± 0.33 **	64.2

* *p* < 0.05, ** *p* < 0.01 *vs.* model control group.

### 2.2. Fucoxanthin Induced Apoptosis in S180 Sarcoma

**Figure 1:**
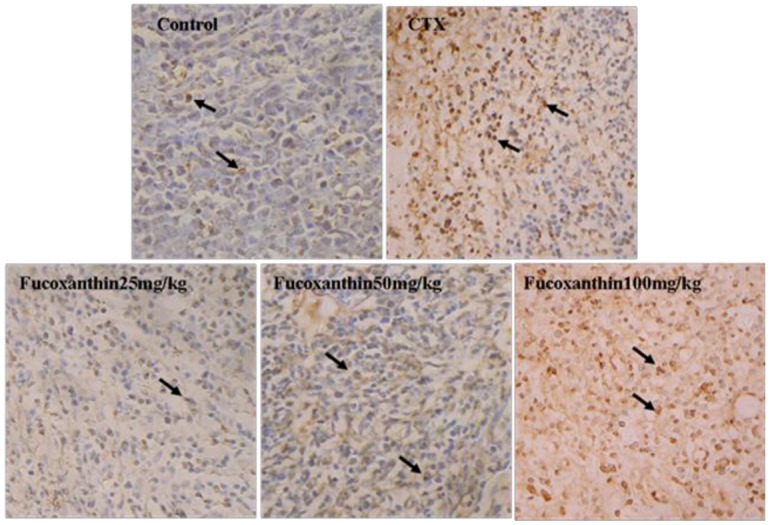
TUNEL analysis for the apoptosis in S180 sarcoma tissue. These sarcoma sections were obtained from the following pretreatment and treatment groups: soybean oil (control), sarcoma 180 (CTX) (20 mg/kg), fucoxanthin at doses of 25 mg/kg, 50 mg/kg and 100 mg/kg. TUNEL-Labeled sarcoma sections showed, following treatment with fucoxanthin (50, 100 mg/kg) and CTX, TUNEL-positive apoptotic cells with brown nuclear staining (black arrow) are significantly greater than those in control (×200). Slides are representative of 15 animals per group.

Apoptosis in sarcoma was detected by *in situ* end-labeling of nuclear DNA fragments (TUNEL) staining. In the TUNEL assay (Figure 1), sarcoma sections from the model control group showed only slight background stained with a few TUNEL-positive cells (5.29 ± 0.28 apoptotic cells/×200 field), whereas in the CTX-treated group, the number of TUNEL-positive cells significantly increased (16.29 ± 0.68 apoptotic cells/×200 field, *p < *0.01, * vs.* model control group). Following treatment with fucoxanthin (50 and 100 mg/kg), the number of TUNEL-positive cells also markedly elevated (9.03 ± 0.58 apoptotic cells/×200 field in 50 mg/kg fucoxanthin group; 17.53 ± 0.62 apoptotic cells/×200 field in 100 mg/kg fucoxanthin group, *p* < 0.05 or *p < *0.01, * vs.* model control group).

### 2.3. The Effects of Fucoxanthin on the Expressions of Cleaved Caspase-3, Bcl-2, Survivin and VEGF

**Figure 2:**
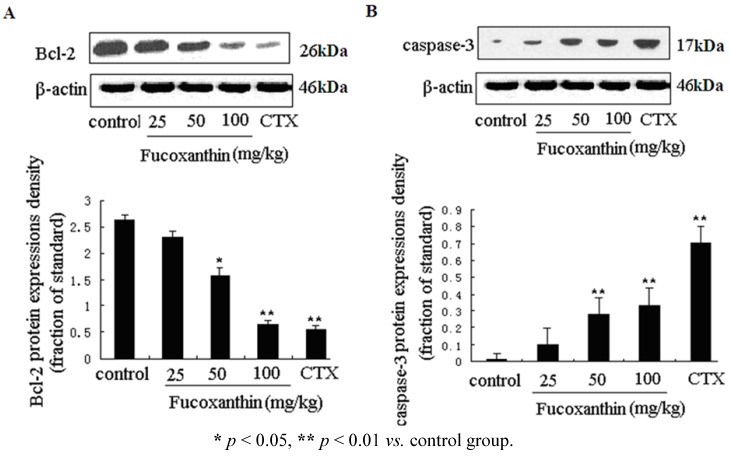
The protein expressions of bcl-2 and cleaved caspase-3 in sarcoma tissue (**A**,**B**). Tissue homogenates from S180 sarcomas were prepared, and then the expression levels of bcl-2 (**A**) and cleaved caspase-3 (**B**) proteins in sarcoma were detected by western blotting. Values are the mean ± SD.

We further investigated the expression levels of two key apoptosis-related genes—caspase-3 and bcl-2 in tumor tissue, which were determined by western blotting (Figure 2A,B). The pro-apoptotic cleaved caspase-3 protein level was obviously increased, while the anti-apoptosis gene bcl-2 protein level was decreased by CTX (*p* < 0.01, *vs.* model control group), fucoxanthin at the dose of 50 and 100 mg/kg (*p* < 0.05 or *p* < 0.01, *vs.* model control group).

**Figure 3:**
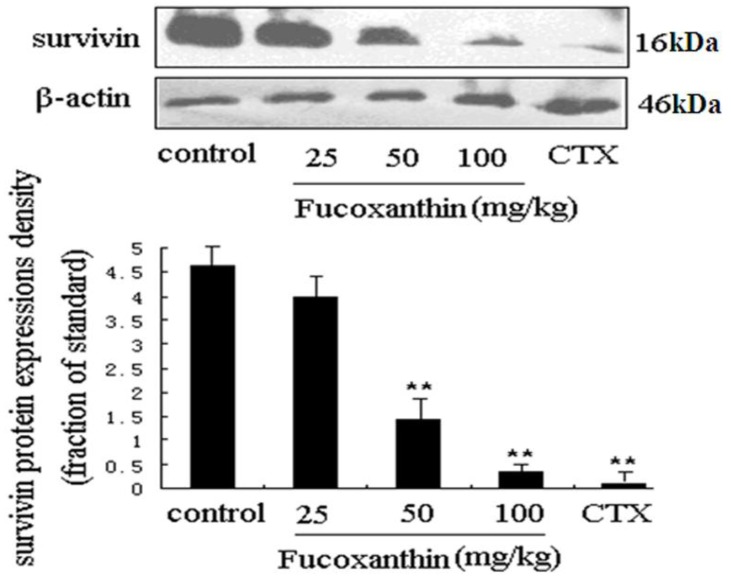
Expression of survivin protein in sarcoma tissue. Western blotting analysis was used to detect the effects of fucoxanthin on the expression of survivin in S180 tumor tissue. The expression of survivin was dramatically decreased by fucoxanthin at the dose of 50 and 100 mg/kg. Values are the mean ± SD. ** *p *< 0.01 *vs.* control group.

**Figure 4:**
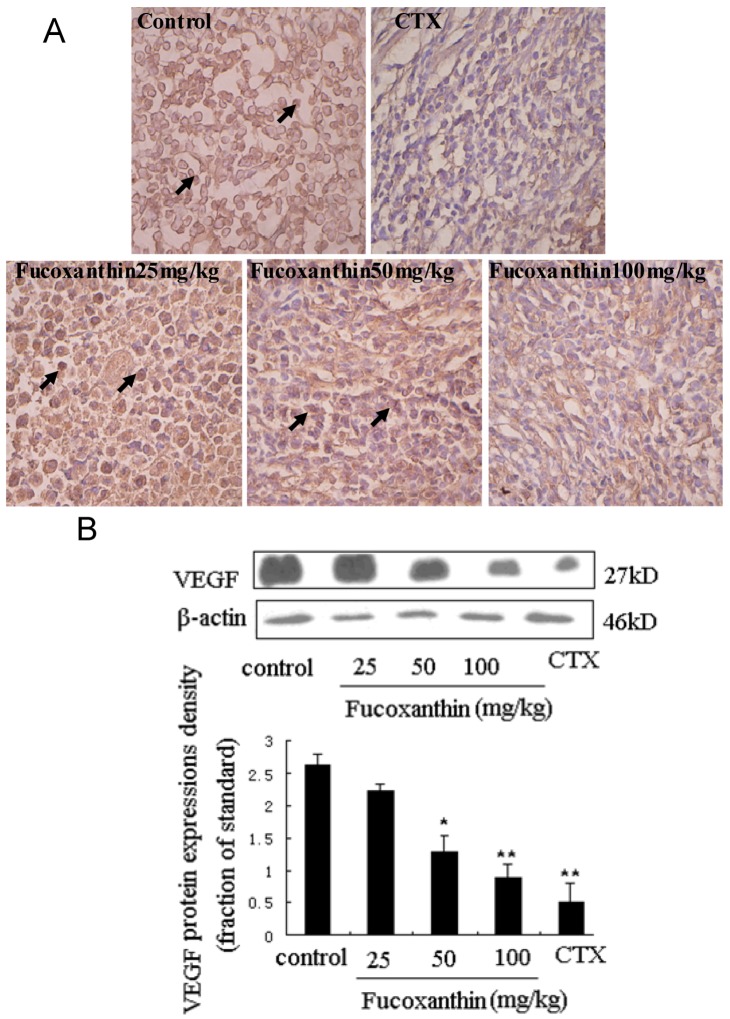
Expression of vascular endothelial growth factor (VEGF) protein in sarcoma tissue (**A**,**B**). (**A**) Sarcoma sections from different groups were immunohistochemically stained to assay the expressions of VEGF protein. The expression of VEGF-positive cells (black arrow) was obviously down-regulated by fucoxanthin (×200). Slides are representative of 15 animals per group; (**B**) Tissue homogenates from S180 sarcomas were prepared, and then the expression levels of VEGF protein in sarcoma were confirmed by western blotting. Western blotting analysis also revealed that the expression of VEGF was decreased by fucoxanthin at the dose of 50 and 100 mg/kg compared with the control group. Values are the mean ± SD. * *p *< 0.05, ** *p* < 0.01 *vs.* control group.

To explore the anti-tumor mechanisms of fucoxanthin in S180 tumor, we examined the protein expressions of survivin and VEGF. Western blotting analysis was used to detect the effects of fucoxanthin on the expression of survivin in S180 tumor tissue. The result showed the expression of survivin was dramatically decreased by CTX and fucoxanthin at the dose of 50 or 100 mg/kg (*p* < 0.05 or *p < *0.01, *vs.* model control group, Figure 3). Immunohistochemistry analysis showed that the expression of VEGF-positive cells were obviously down-regulated by CTX and fucoxanthin (Figure 4A). Western blotting analysis also confirmed that the expression of VEGF (Figure 4B) was decreased by CTX and fucoxanthin at the dose of 50 or 100 mg/kg compared with the control group (*p* < 0.05 or *p < *0.01).

Survivin is the smallest and structurally unique member of the inhibitor of apoptosis (IAP) gene family preferentially expressed in a myriad of clinical cancers [30]. Absent in most adult tissues, surviving is selectively upregulated in many human tumors [31]. The over-expressed survivin protects against apoptosis by either directly or indirectly inhibiting the activation of effector caspases [32]. VEGF is another protein that is a potent stimulator of cancer angiogenesis, inducer of endothelial cell migration and vascular permeability. Notably, VEGF was reported in previous studies to contribute to high degree of vascularization in malignant tumor, where it is up-regulated by oncogenic expression and a variety of growth factors, and promote tumor progression, thus has become a central therapeutic target in treatment of malignancy [33,34]. In our study, western blot assay showed that both survivin and VEGF were overexpressed in model control group but displayed a significantly lower expression in fucoxanthin-treated groups at middle and high dose and CTX-treated group.

### 2.4. The Inhibitive Effects of Fucoxanthin on S180 Sarcoma in Mice Potentially via EGFR/STAT3 Signal Pathway

**Figure 5:**
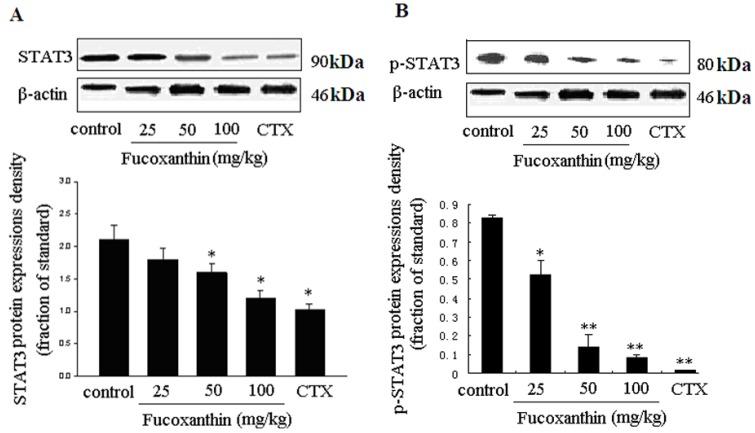
Expressions of STAT3 and p-STAT3 proteins in sarcoma tissue (**A**,**B**). Western blotting analysis was used to detect the effects of fucoxanthin on the expression of STAT3 and p-STAT3 in S180 tumor tissue. At the presence of fucoxanthin at the dose of 50 or 100 mg/kg body weight, the protein expression of STAT3 (**A**) and p-STAT3; (**B**) were reduced than those of the model control group. Values are the mean ± SD. * *p* < 0.05, ** *p* < 0.01 *vs.* control group.

It was reported that survivin and VEGF expressions are both upregulated primarily by activation of STAT3 signaling, which were known as STAT3-survivin signaling pathway [32] and STAT3-mediated VEGF pathway [23,35], respectively. STAT3 is an important member of the STAT family as DNA binding protein. The continuous activation of STAT3 can induce proliferation, differentiation, closely related genes high expression, thus promoting cell proliferation, malignant transformation, blocking cell apoptosis, displaying a strong effect that causes cancer [36]. At present, many STAT3 target genes have been identified including antiapoptotic gene bcl-xl, bcl-2, mcl-1, cyclin D, c-Myc, MMPs, survivin and VEGF [37]. Previous researches have proven that fucoxanthin down-regulated bcl-xl [13] and cyclin D [10,18]. In this research, we found fucoxanthin treatment groups inhibited the expressions of bcl-2, survivin and VEGF. Since these STAT3 target genes all are influenced by fucoxanthin, we raised up a question: could fucoxanthin regulate STAT3 expression in S180 tumor? To address this item, we focused our attention on STAT3 and its phosphorylation protein level. As expected, fucoxanthin decreased the expression of STAT3 and phosphorylated STAT3 (p-STAT3), which was consistent with our *in vitro* result [19]. At the presence of CTX and fucoxanthin at the dose of 50 or 100 mg/kg body weight, the protein expression of STAT3 (Figure 5A) and p-STAT3 (Figure 5B) were reduced than those of the model control group (*p* < 0.05 and *p* < 0.01, respectively), all these effects of fucoxanthin being presented in a dose-dependent manner.

**Figure 6:**
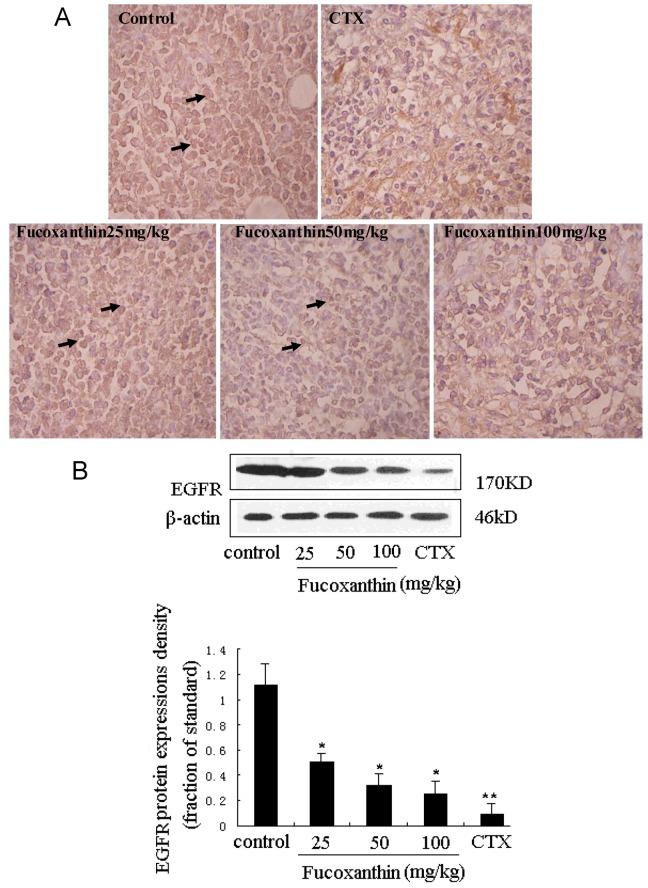
Expression of epidermal growth factor receptor (EGFR) protein in sarcoma tissue (**A**,**B**). (**A**) Sarcoma sections from different groups were immunohistochemically stained to assay the expressions of EGFR protein. The expression of EGFR -positive cells (black arrow) was down-regulated by fucoxanthin (×200). Slides are representative of 15 animals per group; (**B**) Tissue homogenates from S180 sarcomas were prepared, and then the expression levels of EGFR protein in sarcoma were confirmed by western blotting. Treatment with fucoxanthin at the dose of 50 and 100 mg/kg obviously reduced EGFR expression compared with model control group. Values are the mean ± SD. * *p* < 0.05, ** *p* < 0.01 *vs.* control group.

STAT3 has been reported to be activated by interleukin-6 receptor (IL-6R) or EGFR in carcinomas, which may have important implications for responsiveness to therapeutics targeted at EGFR, IL-6R, or intermediary kinases [38]. EGFR pathway is one of the most dysregulated molecular pathways in human cancers. The activation of EGFR, an ubiquitously expressed transmembrane glycoprotein belonging to the ErbB/HER family of receptor tyrosine kinases, has been found to lead to the rapid phosphorylation of STAT3 on tyrosine 705 and the subsequent activation of STAT3-dependent gene expression [39]. On the other hand, activation of the EGFR pathway increases the production of tumor-derived VEGF that acts on endothelial cells in a paracrine manner to promote angiogenesis [40]. EGFR expression was determined by immunohistochemistry and western blot (Figure 6A,B). There were few positive immunostained cells in CTX positive control group, which was also evidenced by western blot. Treatment with fucoxanthin at the dose of 25, 50 and 100 mg/kg obviously reduced EGFR expression compared with the model control group (*p* < 0.05, Figure 6B). These data indicated that, consistent with those of STAT3 and VEGF, the expression of EGFR in the tumor tissue was remarkably declined by treatment with fucoxanthin, suggesting that down-regulation of STAT3/EGFR signaling appeared to involve in the *in vivo *anti-tumor effect and apoptosis induction of fucoxanthin.

## 3. Experimental Section

### 3.1. Chemical

Fucoxanthin was prepared as previous [19], and dissolved in soybean oil to a final concentration of 2.5, 5, 10 mg/mL and stored at 4 °C until used. CTX was purchased from Sigma, USA, and diluted with saline to a concentration of 2 mg/mL.

### 3.2. Animal Models and Treatment Protocols

Male Kunming mice (6–8 weeks old, 20 ± 2 g) were obtained from the Experimental Animal Center of the Hubei Province (Grate II, Certificate No. SCXK-2008-0004, Hubei, China). S180 tumor cells used in experiment was given by Tongji Medical College of Huazhong University of Science and Technology. Mice were housed at a constant room temperature of 22 °C and maintained under controlled conditions of 12 h light/12 h dark photoperiod. All animals received human care according to the criteria outlined in the Guide for the Care and Use of Laboratory Animals published by the National Institutes of Health, and the experiments were approved by the Animals Care and Use Committee of Wuhan University of Science and Technology Medicine College. After fed in our facility for 1 week, all 75 mice were randomly divided into 5 groups of 15 animals in each group: (1–3) fucoxanthin treatment groups: administered with fucoxanthin, at low (25 mg/kg), middle (50 mg/kg), and high (100 mg/kg) oral administration dose once per day respectively; (4) model control: orally administered with the same volume of soybean oil; (5) positive control group: CTX was injected intraperitoneally at a dosage of 20 mg/kg body weight once per day. After fucoxanthin pretreatment for 1 week, sarcoma 180 ascites tumor cells (2 × 10^6^ cell/500 μL) were implanted subcutaneously into the left hind groin of the mice in all groups. 24 h after inoculation, all animals were treated as planned. On day 14 after inoculation, all animals were sacrificed, and then the sarcoma tissues were isolated and weighted for TUNEL, immunohistochemistry and western blot analysis.

### 3.3. Estimation of DNA Fragmentation

Small pieces of sarcoma tissues taken from experimental animals were fixed in 10% formalin, dehydrated with alcohol, embedded in paraffin, and sectioned to a mean thickness of 4 μm. Then, the sections were used to perform TUNEL. Briefly, the tissue sections were hydrated with gradient alcohol (100, 95, 90, 80, 70%). The endogenous peroxidase activity was blocked using 3% hydrogen peroxide in methanol for 3 min at 20 °C. After washing with PBS, the sections were permeabilized with Proteinase K solution (4 mg Proteinase K powder dissolved in 200 mL 10 mMTris/HCl to a final concentration of 20 μg/mL, pH = 7.4–8.0) for 1 min on the ice. After permeabilization, the sections were exposed to the mixture of TdT and dUTP at 37 °C for 60 min. A converter-POD solution was applied to the sections for 30 min at 37 °C in darkness. And then the sections were stained with DAB to generate a brown reaction product. Negative controls were performed at the same time. The number of positive cells was counted using the light microscope.

### 3.4. Immunohistochemical Examination

The paraffin-embedded sections were placed for 60 min at room temperature. Then the paraffin sections were dewaxed in xylene for 10 min, rehydrated with a series of gradient alcohol (100, 95, 90, 80, 70%), respectively. After that the sections were incubated in methanol containing 3% H_2_O_2_ for 10 min to inactivate endogenous peroxidase. Sections were incubated with Clean Vision™ blocking solution (ImmunoVision Technologies Co., CA, USA) for 1–2 h. Following washing three times for 5 min in PBS, the slides were incubated with the flowing antibody: rabbit anti-EGFR polyclonal antibody or mouse anti-VEGF monoclonal antibody (ab2430 abcam, ab1316 abcam, UK) at a dilution of 1:800 and 1:1000, respectively at room temperature for 2 h. And then a biotinylated goat anti-rabbit or goat anti-mouse antibody (Santa Cruz, CA, USA) was applied as secondary antibodies at room temperature for 2 h. Peroxidase activity was revealed by dipping the sections in a mixture containing 0.05% 3,3′-diaminobenzidine (DAB) and 0.03% H_2_O_2_ for 5 min. The sections were then counterstained with hematoxylin, coverslipped, and observed under a microscope.

### 3.5. Western Blotting Analysis

The sarcoma tissues were removed from mice in each group and then washed twice with cold PBS before homogenized with a homogenizer in ProteoJET™ Mammalian Cell Lysis Reagent (MBI fermentas) followed by centrifugating at 4 °C, 14,000 rpm for 20 min. The protein concentration in the lysate was measured by the Lowry method [41]. The supernatants (50 µg protein/lane) were separated by 10% SDS-PAGE and then transferred to nitrocellulose membrane by electroblotting. The membrane was then blocked with TBS-T (20 mM Tris–HCl (pH 7.6), 137 mM NaCl, and 0.1% Tween 20) containing 5% nonfat dry milk for 1 h at room temperature. The sources of primary antibodies used in this study were as follows: VEGF, EGFR (ab1316 abcam, ab2430 abcam, UK), survivin, bcl-2, caspase-3, STAT3, p-STAT3 (tyrosine 705), β-actin (sc-47750, sc-7382, sc-56055, sc-8019, sc-8059, sc-47778, Santa Cruz, CA, USA). The membrane was incubated with above antibodies for 2 h. After washing with 0.1% Tween-20 in TBS, the membranes were incubated with a secondary antibody horseradish peroxidase-conjugated anti-mouse IgG/anti-rabbit IgG (Santa Cruz, CA, USA) for 2 h at room temperature. Finally, the membrane was treated with the reagents in the enhanced chemiluminescence detection kit (ECL system, Amersham Pharmacia Biotech, Piscataway, NJ, USA) according to the manufacturer’s instructions and exposed by using an X-ray film. The relative density of the immunoreactive bands was quantitated by Quantity One (Version 4.6.2, Bio-Rad Technical Service Department, USA). The relative amount of survivin, VEGF, caspase-3, bcl-2, EGFR, STAT3 and p-STAT3, in each lane was obtained after correcting with the amount of β-actin in the same sample.

### 3.6. Statistical Analysis

All the grouped data were statistically evaluated with SPSS11 software. Hypothesis testing methods included one way analysis of variance (ANOVA) followed by least significant difference (LSD) test; *p* value of less than 0.05 were considered to indicate statistical significance. All the results were expressed as the mean ± SD for 15 animals in each group.

## 4. Conclusions

In summary, our results suggest that fucoxanthin has *in vivo* anti-tumor effect and its mechanism is attributed to induced apoptosis. *In vivo*, Fucoxanthin significantly upregulated the expression of pro-apoptotic cleaved caspase-3, decreased the expressions of bcl-2, EGFR, STAT3, survivin and VEGF in a dose-dependent manner, which are EGFR/STAT signal pathway-related genes. These results indicated that *in vivo* induction of apoptosis by fucoxanthin is associated with down-regulating STAT3/EGFR signaling in S180 xenografts-bearing mice.
